# The Effects of Exercise Training on Body Composition and Cardiometabolic Risk Factors in Type 1 Diabetes Mellitus: A Systematic Review and Meta-Analysis

**DOI:** 10.3390/healthcare13030246

**Published:** 2025-01-26

**Authors:** Mousa Khalafi, Farnaz Dinizadeh, Sara K. Rosenkranz, Michael E. Symonds, Saeid Fatolahi

**Affiliations:** 1 Department of Sport Sciences, Faculty of Humanities, University of Kashan, Kashan 87317-53153, Iran; 2 Department of Sport Sciences, Tabriz Branch, Azad University, Tabriz 51579-44533, Iran; farnazdinizadeh@gmail.com; 3 Department of Kinesiology and Nutrition Sciences, University of Nevada Las Vegas, Las Vegas, NV 89154, USA; sara.rosenkranz@unlv.edu; 4 Centre for Perinatal Research, Academic Unit of Population and Lifespan Sciences, School of Medicine, University of Nottingham, Nottingham NG7 2UH, UK; michael.symonds@nottingham.ac.uk; 5 Department of Physical Education and Sport Sciences, Faculty of Humanities, Tarbiat Modares University, Tehran 14117-13116, Iran

**Keywords:** type 1 diabetes, exercise, training, body composition, cardiometabolic risk factors

## Abstract

Introduction and Aim: We performed a systematic review and meta-analysis to investigate the effects of exercise training on body composition and cardiometabolic health in patients with Type 1 diabetes (T1D). Method: A search in three main databases including PubMed, Web of Science, and Scopus was conducted from the inception of this review until June 2024 to identify randomized control trials investigating the effects of exercise training compared to a control on body composition and cardiometabolic risk factors in patients with T1D. The data were pooled using random effects models to calculate weighted mean differences (WMDs), standardized mean differences (SMDs), and 95% confidence intervals (CIs). Results: Overall, 25 studies involving 1120 patients with T1D were included in the meta-analysis. Exercise training decreased body mass index (BMI) [WMD: −0.18 kg.m^2^, *p* = 0.02], fasting glucose [WMD: −14.97 mg/dl, *p* = 0.01], and HbA1c [WMD: −0.49%, *p* = 0.003], and increased VO_2max/peak_ [WMD: 2.76 mL/kg/min, *p* = 0.001] as compared with controls. Exercise training had no effect on body fat percentage or lean body mass, lipid profiles, or blood pressure. Subgroup analysis indicated that age, exercise mode, and intervention duration were the main moderators for the beneficial effects of exercise training. Conclusions: In patients with T1D, exercise training is effective for decreasing body weight and cardiometabolic risk factors.

## 1. Introduction

Type 1 diabetes mellitus (T1D) is a chronic autoimmune disease caused by the immune-mediated destruction of beta cells that is characterized by hyperglycemia and insulin deficiency [1,2]. The incidence and prevalence of T1D has increased globally over the past few decades and T1D is associated with increased risk of cardiovascular diseases irrespective of age [2,3,4,5,6]. Management approaches aim to reduce cardiometabolic risk factors, including with improved glycemic control, lipid profiles, blood pressure, and body composition, thereby preventing or delaying the onset of T1D-associated complications [7,8,9,10].

Regular exercise training is recommended for individuals with metabolic derangements and diseases. Research has shown that exercise training can provide multiple health benefits, including improved body composition and cardiometabolic risk factors such as lipid profiles, glycemic control, and blood pressure. Although specific recommendations and precautions for exercise training in patients with T1D depend on individual characteristics and health statuses, 150 min or more of moderate-to-vigorous aerobic activity (AET) spread across a minimum of 3 days/week, with no more than 2 consecutive non-activity days, and 2–3 sessions/week of resistance training (RET) on non-consecutive days is recommended [11]. For people with T1D, shorter durations (minimum of 75 min/week of vigorous-intensity activity or interval training may be sufficient. For people with T1D, despite the previously reported beneficial effects of exercise training on skeletal muscle mass, muscle function, body composition, fitness, lipid profiles, glycemic markers, and oxidative capacity [12,13], exercise management remains challenging. Results from randomized trials [14,15,16,17,18,19,20,21] and previous meta-analyses are also mixed, indicating a need for elucidating the role of exercise training in patients with T1D [22,23,24,25,26]. Potential reasons for these inconsistencies include the modes and durations of exercise interventions, the quality of studies included, and potentially the role that participant age plays. Another reason for conducting the present meta-analysis is the limitations of some previous publications, such as the inclusion of non-randomized studies on the effect of regular exercise on people with T1D [27,28,29]. Recent research has highlighted that including non-randomized intervention studies in meta-analysis of randomized controlled trials may introduce a higher risk of bias in effectiveness estimates compared to those derived only from randomized controlled trials [30]. Moreover, previous meta-analyses [22,28,29] were not confined to individuals with T1D. Therefore, the aim of the present meta-analysis is to investigate the comprehensive effects of exercise training on body composition and cardiometabolic risk factors including glycemic markers, lipid profiles, blood pressure, and cardiorespiratory fitness in individuals with T1D. Further we aimed to determine the potential moderating effects of exercise modes, intervention durations, and participant ages.

## 2. Methods

This systematic review and meta-analysis were performed in accordance with the Preferred Reporting Items for Systematic Reviews and Meta-analysis (PRISMA) guidelines and the Cochrane Handbook for Systematic Reviews of Interventions. The systematic review and meta-analysis were registered at the International Prospective Register of Systematic Reviews (PROSPERO) with the following registration: CRD42024593149.

### 2.1. Search Strategy

Comprehensive searches were conducted in the three main electronic databases, including PubMed, Web of Science, and Scopus, from inception through June 2024. Searches were performed using three primary key words, including (exercise OR “exercise training” OR “physical activity”) AND (“Type1 diabetes” OR “Insulin-Dependent” OR “Insulin-Dependent Diabetes Mellitus” OR “Type 1 Diabetes Mellitus” OR “Insulin-Dependent Diabetes Mellitus 1” OR IDDM)) AND (random OR randomized OR randomly OR “randomized control”. Filters were used, where possible, to identify only studies of humans and articles published in the English language. In addition, the reference lists of included studies, previous relevant meta-analysis, and Google Scholar were manually searched for additional studies not identified in the original searches. The details of the search strategy are provided in Table S1. Two authors (M.K. and S.F.) independently conducted the main and additional searches.

### 2.2. Identification and Selection Criteria

All retrieved records were exported to EndNote (version 20.2.1) where duplicate records were removed. Subsequently, the titles and abstracts, and then the full texts of eligible studies, were screened against the inclusion and exclusion criteria. Only studies published in peer-reviewed journals were considered based on a priori PICOS criteria (participants, intervention, comparator, outcome, study design). Population: human subjects diagnosed with T1D, regardless of age, biological sex, and health status; intervention: exercise training with a minimum duration of two weeks, regardless of mode, intensity, and frequency; comparator: non-exercise control groups; outcomes: results for at least one of the following outcomes including body weight, fat mass, body mass index (BMI), body fat percentage (BFP), fat-free mass (FFM) (or lean body mass), waist circumference (WC), fasting glucose, fasting insulin, glycated hemoglobin A1c (HbA1c), total cholesterol (TC), low-density lipoprotein (LDL), high-density lipoprotein (HDL), triglycerides (TG), systolic blood pressure (SBP), diastolic blood pressure (DBP), or VO_2max/peak_; study design: intervention and control groups with randomized parallel or crossover designs. The following exclusion criteria were applied: non-original studies, studies involving participants with type 2 diabetes mellitus, and non-randomized control trials. Study selection and screening processes were performed by two independent authors (S.F. and F.S.) and any disagreements were resolved through discussion with another author (M.K.).

### 2.3. Data Extraction and Synthesis

The following information and data were extracted from the included articles: first author name and publication year, sample size, study design, participant characteristics including age, sex, and BMI, intervention characteristics including exercise mode, exercise intervention duration and protocol, and outcome variables. In addition, to perform the meta-analysis, mean changes (post–pre values) and their standard deviations (SD) were extracted or calculated from the mean and SD values at pre- and post-intervention using a formula recommended in the Cochrane Handbook with conservative values of 0.5 for correlations. When data were reported as medians and IQRs, or standard errors and 95% confidence intervals (CIs), the required data were calculated using validated methods [31,32,33]. In addition, when data were only reported in figures, the required data were extracted using the Getdata Graph Digitizer software (2.24). If studies had more than one exercise intervention arm, they were included as separate arms and the sample size for the control group was halved. In order to enhance the clinical interpretation of the results, the data for lipid profiles and fasting glucose were expressed as mg/dL, and, when required, mmol/L values were converted to mg/dL using relevant formulas [34]. In addition, for HbA1c, units reported in mmol/mol were converted to percentages using the relevant formula [35]. The data were extracted by two independent authors (S.F. and F.S.) and any disagreements were resolved through discussion with another author (M.K.).

### 2.4. Quality Assessment

Study quality was determined using the Physiotherapy Evidence Database (PEDro) scale, which assesses the risks of bias using 11 standard items. However, two items, including blinding of participants, and blinding of all therapists were excluded, due to the impossibility of this type of blinding in exercise trials. Finally, the overall quality of the included studies was assessed using 9 items, with a higher score indicating a lower risk of bias and higher quality for the study (Table S2). The quality assessment was conducted by two independent authors (S.F. and F.S.) and any disagreements were resolved through discussion with another author (M.K.).

### 2.5. Statistical Analysis

A meta-analysis was performed to compare the effects of exercise training versus non-exercise control groups when there were at least 3 interventions for each outcome. Data were pooled using random effects models and standardized mean differences (SMD) when measurement units were different between studies, or weighted mean differences (WMD) and 95% CIs when measurement units were the same. To examine the heterogeneity amongst included studies, the Cochrane Q test and I^2^ statistics were calculated. The I^2^ values were interpreted according to Cochrane guidelines, with 25%, 50%, and 75% indicating low, moderate, and high heterogeneity, respectively. To examine the potential for publication bias, visual interpretation of funnel plots was used. In addition, Egger’s tests were used to determine the presence of publication bias as a secondary determinant when *p*-values were <0.10. A sensitivity analysis was then performed by removing individual studies to ensure that the results were not significantly affected by a single study. Sensitivity analysis was performed when there were more than 10 intervention arms. Several subgroup analyses were performed based on age (children/adolescents: <18 years, and adults: ≥18 years), exercise mode (AET, RET, combined (CET), and high-intensity interval training (HIIT)), as well as intervention duration (medium-term: <16 weeks and longer-term: ≥16 weeks). All analysis, funnel plots, and forest plots were completed using the Comprehensive Meta-analysis (CMA) software version 3.

## 3. Results

### 3.1. Search Strategy

**Figure 1:**
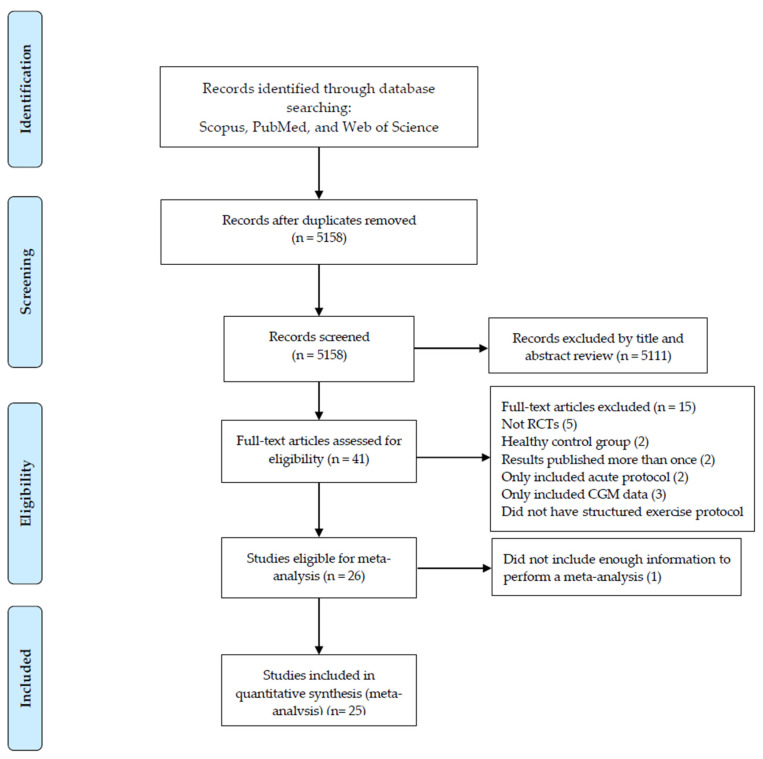
Flow diagram of systematic literature search.

The three main electronic database searches yielded 5532 records and 5158 records remained after removing duplicates. Based on the title and abstract screening, 5117 records were excluded, and, subsequently, 41 articles were eligible for the full-text screening. Finally, 16 articles were excluded for reasons presented in Figure 1, and 25 articles met all inclusion criteria and were included in the meta-analysis. All included studies were randomized control trials with parallel or crossover designs.

### 3.2. Study Characteristics

A total of 25 studies, comprising 30 intervention arms and 1120 participants were included in the meta-analysis. Sample sizes ranged from 10 to 20, ages ranged from 10 to 60 years, and BMIs ranged from 24 to 32 kg.m^2^. Ten studies included only males, eleven studies included males and females, and four studies included only females as participants. Exercise training modes included AET, RET, combined (CET), and HIIT. Intervention durations ranged from 4 weeks to 12 months, and exercise session frequency ranged from 3 to 5 sessions/week. The details regarding participants and exercise intervention characteristics, and overall study quality, are presented in Table 1.

### 3.3. Meta-Analysis

#### 3.3.1. Body Composition

**Figure 2:**
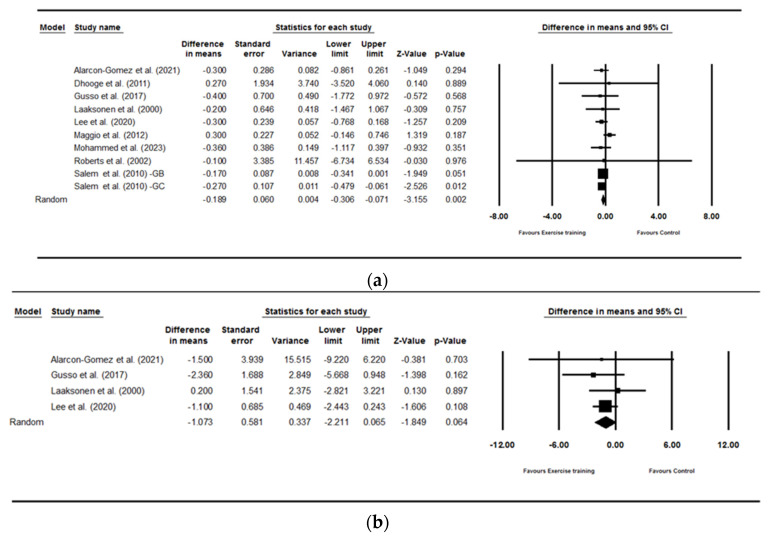

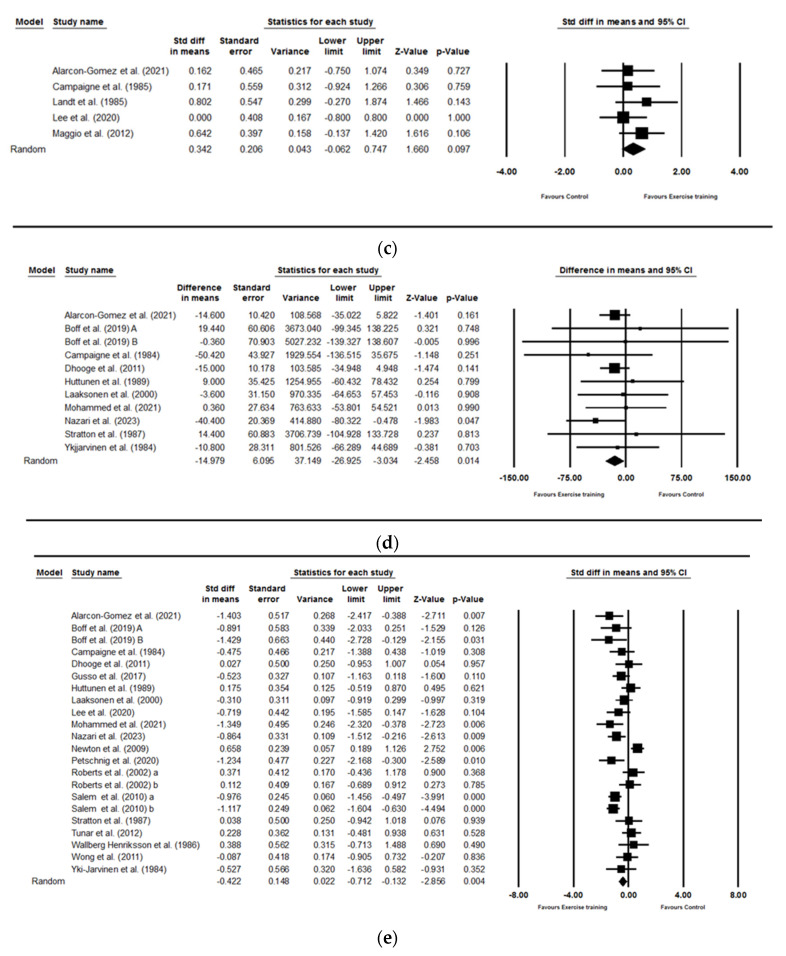
Forest plot of the effects of exercise training versus control on BMI (**a**), BFP (**b**), LBM (**c**), fasting glucose (**d**) and HbA1c (**e**). Data are reported as WMD (95% confidence limits). WMD: weighted mean difference.

Exercise training decreased BMI significantly more than controls [WMD: −0.18 kg.m^2^ (95% CI: −0.30 to −0.07), *p* = 0.02; 9 studies]. However, exercise training did not change BFP [WMD: −1.07% (95% CI: −2.21 to 0.06), *p* = 0.06; 4 studies] or LBM [WMD: 0.61 kg (95% CI: −0.37 to 1.59), *p* = 22; 5 studies] when compared with controls (Figure 2). This reduction in BMI suggests that exercise training primarily influences overall body fat reduction, though it does not significantly affect fat BFP or LBM. There was no significant heterogeneity amongst studies for BMI (I^2^ = 0.00, *p* = 0.74), BFP (I^2^ = 0.00, *p* = 0.73), or LBM (I^2^ = 0.00, *p* = 0.43). The visual interpretation of funnel plots suggested publication bias, but Egger’s test results did not confirm publication bias for BMI (*p* = 0.89), BFP (*p* = 0.92), or LBM (*p* = 0.25). The analytical interpretation of these results is presented in the discussion section.

#### 3.3.2. Glycemic Markers

Exercise training decreased fasting glucose [WMD: −14.97 mg/dL (95% CI: −26.92 to −3.03), *p* = 0.01; 10 studies] and HbA1c significantly more than controls [WMD: −0.49% (95% CI: −0.82 to −0.16), *p* = 0.003; 19 studies] (Figure 2). These reductions in fasting glucose and HbA1c indicate that exercise training may improve glycemic control, which is critical for managing diabetes and reducing long-term complications in T1D. There was significant heterogeneity amongst included studies for HbA1c (I^2^ = 74.91, *p* = 0.001), but not for fasting glucose (I^2^ = 0.00, *p* = 0.95). The visual interpretation of funnel plots suggested publication bias, but the Egger’s test results only confirmed this bias for HbA1c (*p* = 0.06), not for fasting glucose (*p* = 0.42). The discussion section offers a deeper insight into the inferential analysis of these results.

#### 3.3.3. Lipid Profiles

Exercise training did not decrease TG [WMD: −5.37 mg/dL (95% CI: −25.04 to 14.28), *p* = 0.59; 11 studies], TC [WMD: −11.82 mg/dL (95% CI: −25.38 to 1.73), *p* = 0.08; 11 studies], LDL [WMD: −4.88 mg/dL (95% CI: −15.78 to 6.00), *p* = 0.37; 9 studies], or HDL [WMD: 1.50 mg/dL (95% CI: −3.28 to 6.29), *p* = 0.53; 11 studies] significantly more than control groups (Figures S1–S4). These findings suggest that exercise training had minimal impact on lipid profiles, indicating that exercise alone may not be sufficient to induce significant changes in TG, TC, LDL, or HDL levels in patients with T1D. There was significant heterogeneity amongst the included studies for TG (I^2^ = 83.42, *p* = 0.001), TC (I^2^ = 72.88, *p* = 0.001), LDL (I^2^ = 64.52, *p* = 0.002), and HDL (I^2^ = 77.05, *p* = 0.001). The visual interpretation of funnel plots suggested publication bias, but the Egger’s test results confirmed publication bias for TC (*p* = 0.09) and HDL (*p* = 0.001), but not for TG (*p* = 0.51). In addition, both visual interpretation of funnel plots and Egger’s test results did not suggest publication bias for LDL (*p* = 0.49). The discussion section elaborates on the inferential analysis of these results.

#### 3.3.4. Blood Pressure

Exercise training did not decrease SBP [WMD: 1.26 mmHg (95% CI: −1.81 to 4.34), *p* = 0.42; 4 studies] or DBP [WMD: 1.11 mmHg (95% CI: −1.29 to 3.52), *p* = 0.36; 4 studies] significantly more than control groups (Figures S5 and S6). These findings highlight that exercise training did not lead to significant improvement in BP, indicating that exercise alone may not be sufficient to induce meaningful changes in SBP or DBP in this population. There was no significant heterogeneity amongst included studies for SBP (I^2^ = 0.00, *p* = 0.50) or DBP (I^2^ = 0.00, *p* = 0.64). The visual interpretation of funnel plots only suggested publication bias for SBP, but the Egger’s test results confirmed this bias for both SBP (*p* = 0.88) and DBP (*p* = 0.78), suggesting that they may be influenced by publication bias. The reasoning behind these results is presented in the discussion section.

#### 3.3.5. VO_2max/peck_

Exercise training increased VO_2max/peck_ [WMD: 2.76 mL/kg/min (95% CI: 1.40 to 4.13), *p* = 0.001; 9 studies] significantly more than what was seen in control groups (Figure S7). This improvement in VO_2max/peck_ suggests that exercise training effectively enhances aerobic capacity, which is crucial for cardiovascular health and overall physical fitness in people with T1D. There was no significant heterogeneity amongst included studies (I^2^ = 27.88, *p* = 0.17). Neither the visual interpretation of funnel plots nor the Egger’s test results suggested publication bias for VO_2max/peak_ (*p* = 0.70). The discussion section includes the interpretation of these results.

### 3.4. Subgroup Analysis

#### 3.4.1. Body Composition

BMI was decreased by CET training WMD [−0.21 kg, *p* = 0.002; four studies], in children/adolescents [−0.17 kg, *p* = 0.006; six studies].

#### 3.4.2. Glycemic Markers

HbA1c was reduced by CET [WMD: −0.94%, *p* = 0.002; 4 studies] and longer-term interventions [WMD: −0.86%, *p* = 0.01; 5 studies], and in children/adolescents [WMD: −0.45%, *p* = 0.04; 14 studies] and adults [WMD: −0.52%, *p* = 0.01; 6 studies]. Fasting glucose was reduced in children/adolescents [WMD: −17.16 mg/dL, *p* = 0.03; six studies].

#### 3.4.3. Lipid Profiles

TC was decreased by AET [WMD: −16.72 mg/dL, *p* = 0.02; five studies]. TG was decreased by CET [WMD: −22.10 mg/dL, *p* = 0.04; four studies]. HDL was increased by CET [WMD: 10.59 mg/dL, *p* = 0.001; three studies], longer-term interventions [WMD: 10.81 mg/dL, *p* = 0.001; three studies], and in children/adolescents [WMD: 8.77 mg/dL, *p* = 0.001; six studies].

#### 3.4.4. VO_2max/peak_

VO_2max/peak_ was increased by AET [WMD: 2.53 mL/kg/min, *p* = 0.007; seven studies] and HIIT [WMD: 3.96 mL/kg/min, *p* = 0.01; three studies], and in adults [WMD: 3.48 mL/kg/min, *p* = 0.001; five studies]. The results of the inferential analysis related to the subgroups are reported in the discussion section. 

## 4. Discussion

This systematic review and meta-analysis included 25 RCTs of T1D patients demonstrating that, when compared with control groups, exercise training interventions are effective for improving body composition and cardiometabolic risk factors. More specifically, exercise training was effective for decreasing BMI, HbA1c, and fasting glucose and for increasing VO_2max/peak_. However, exercise training did not reduce BFP, LBM, lipid profiles, or BP more than control groups. Further subgroup analysis indicated beneficial effects of combination exercise training (CET) for reducing BMI, HbA1c, and HDL, while AET was effective for reducing TC and increasing VO_2max/peak_. Furthermore, longer-term training (more than 16 weeks) improved HbA1c and HDL. Exercise training in children and adolescents with T1D, was beneficial for reducing BMI, HbA1c, fasting glucose, and HDL, whilst HIIT enhanced VO_2max/peak_, for subgroups ≥ 18 years old.

### 4.1. Body Composition

A healthy weight range, determined by BMI, and optimal body composition are crucial factors in determining health-related risk in patients with T1D [55,56,57], and several meta-analysis have indicated significant benefits for exercise training [25,27,58]. In line with our findings, one meta-analysis included 15 randomized and/or controlled trials with 596 total participants, and demonstrated that exercise training durations of ≥12 weeks reduced BMI in adults [25], whereas another meta-analysis with 4 studies and 195 participants indicated that exercise training reduced BMI effectively [58]. Conversely, a meta-analysis comprising 24 RCTs, quasi-experimental trials, and crossover trials, including 998 individuals with T1D, revealed no effects following various exercise training modalities, for more than 4 weeks, on BMI [27]. These contradictory results can be attributed to the type and number of studies included in the meta-analysis. As it presently seems, the effectiveness of CET in children under 18 years old was likely attributable to the long duration of training (more than 16 weeks). To the best of our knowledge, no meta-analysis has investigated the effects of exercise training, BFP, or LBM on people with T1D. Previous studies have shown the ineffectiveness of RCTs with CET and HIIT modalities on BFP in T1D patients between trained and control groups [14,18,43,52]. However, despite the potential effect of lowering the required insulin dose through exercise training, which may indirectly prevent fat accumulation in patients with T1D [13,59], prescribing an optimal diet with exercise training is an important strategy. In T1D patients, LBM alterations are mixed [14,18,38,39,50], with the exercise type being critical.

### 4.2. Glycemic Markers

In T1D, HbA1c improvement is strongly associated with a reduction in peripheral vascular, metabolic, and cardiovascular disease risks, nephropathy, and neuropathy [60,61]. A 1% reduction in HbA1c is linked to a 37% decrease in the risk of microvascular complications, a 14% reduction in the risk of acute myocardial infarction, and a 21% lower risk of death from T1D [62]. Several meta-analyses including various study designs have investigated the effects of exercise on HbA1c in patients with T1D [22,27,29], with one that included 14 RCTs involving 509 children and adolescents. Consistent with our findings, the meta-analysis demonstrated a reduction in HbA1c following concurrent exercise training [22]. Similar results from another meta-analysis indicated exercise training in participants with T1D-lowered HbA1c [58]. Contrary to the results of our study, a separate meta-analysis that included 12 randomized and non-randomized studies in adults and children with T1D showed no effect of exercise training on HbA1c [29]. The present study, with strict inclusion criteria that selected only RCTs, demonstrated a greater improvement in HbA1c in children and adolescents under 18 years of age who engaged in CET for 16 weeks or longer, and is in accord with a meta-analysis by García-Hermoso and colleagues (2023). The meta-analysis revealed that exercise for longer than 60 min, maintained for more than 24 weeks, substantially improved HbA [22]. Furthermore, in patients with T1D who were under 18 years of age, exercise interventions lasting more than 12 weeks also had a significant effect [27]. However, a separate meta-analysis did not indicate any difference in effectiveness between interventions with durations longer or shorter than 12 weeks. However, the meta-analysis did indicate that three or more weekly sessions had beneficial effects as compared with less than three sessions per week [58]. These findings suggest that CET for at least 12 weeks can have beneficial effects on HbA1c levels, with children and adolescents potentially being more responsive than adults. Similarly to HbA1c outcomes, fasting glucose levels were effectively reduced following exercise training when compared with control groups, and this was a consistently reported effect, particularly in children and adolescents. While fasting glucose is not the gold standard for measuring (fasting glucose) control in patients with T1D, the substantive change in fasting glucose indicates better (FBG) regulation overall [63,64].

### 4.3. Lipid Profiles

Patients diagnosed with T1D may have lipid abnormalities associated with cardiovascular complications [65], and lipid profiles are also related to glycemic control and metabolic complications [66,67]. A previous meta-analysis indicated that exercise training improves TC by ~0.38 mmol/L, but other lipid profile markers were unchanged [27] which was confirmed by another meta-analysis from Quik and colleagues (2014) that observed positive effects on AET or CET on TC (SMD = −0.91) and TG (SMD = −0.70) in children and adolescents with Type 1 diabetes [58]. In our pooled analysis of lipid profiles, some discrepancies in these results can be attributed to the varying inclusion criteria of the studies included in the meta-analysis and the presence or absence of dyslipidemia in participants at baseline. Higher HDL levels are associated with lower risk of cardiovascular diseases, and studies have suggested that HDL-related therapeutic targets in T1D might provide a valuable complementary therapy [68,69,70]. Recently, some research has suggested that, rather than focusing on the levels of HDL per se, the function of HDL is an important factor to consider. Some recent evidence from a large cohort study (UK Biobank) actually indicates that very high HDL levels are associated with a higher risk of mortality in participants with coronary artery disease [71]. It is not clear whether high HDL levels would be detrimental in patients with T1D.

### 4.4. Blood Pressure

Hypertension is a risk factor for cardiovascular diseases, and is prevalent in patients with T1D [72], with 80% experiencing microalbuminuria and diabetes-induced nephropathy, which play an important role in the pathogenesis of hypertension [73]. Previous meta-analyses have shown that exercise training can improve blood pressure levels in patients with hypertension [74] and T2D [75]. Consistently, Wu and colleagues (2019) supported the present study’s findings, which confirmed no effect of exercise training on SBP or DBP in patients with T1D [27]. Possible explanations include low baseline resting blood pressures in participants, and insufficient levels of vigorous or high-intensity exercise training. Although blood pressure is important in T1D patients, evidence for effective strategies for reducing blood pressure in this population requires further investigation.

### 4.5. VO_2max/peak_

Cardiorespiratory fitness (CRF) is a strong predictor of all-cause mortality, and VO_2max_ is a primary determinant [76,77]. A recent meta-analysis reported that patients 18 years of age or younger with T1D exhibited a 10% decrease in CRF [78] compared to those without diabetes. A meta-analysis of 11 studies with a variety of included study designs confirmed that exercise training significantly increased CRF in T1D participants, with three or more sessions per week and a duration of longer than 12 weeks and involving AET [27]. A separate meta-analysis also found a moderate improvement in CRF in adolescents with T1D, with a mean increase of 3.94 mL/kg/min following concurrent exercise training [22]. Our findings indicated that relative VO_2max/peak_ was increased following AET as well as HIIT. In the current investigation, it is of note that VO_2max/peak_ improved in adults with T1D following AET protocols, but did not improve in children and adolescents. This lack of improvement can be explained by interventions that did not include sufficient intensities or durations of exercise training. Aerobic exercise is significantly correlated with reduced cardiovascular and total mortality risks [79], with HIIT potentially lowering fasting glucose, thus providing an effective exercise intervention in patients with T1D [28]. According to current and previous meta-analyses, to improve VO_2max/peak_ in patients with T1D, healthcare providers should prescribe AET or HIIT with a weekly frequency of three or more sessions and a minimum duration of 12 weeks.

### 4.6. Limitations

The current meta-analysis had several limitations that should be considered when interpreting the results. First, there were only a small number of studies for several outcomes that prevented a more comprehensive analysis. There was high heterogeneity amongst all included studies that may be explained by the range of ages, exercise modes, and intervention durations. In addition, management of T1D requires long-term therapeutic interventions, while most of the included studies only used short-term exercise training.

## 5. Conclusions

The findings of the current meta-analysis suggest that exercise training improves body composition and cardiometabolic risk factors in patients with T1D. Specifically, it reduces BMI and glycemic markers while enhancing VO_2max/peak_, though it has no significant impact on body composition, lipid profiles, or blood pressure. Also, findings highlight that CET effectively improved BMI, HbA1c, and HDL, while AET lowered TCH and enhanced aerobic capacity. Exercise for more than 16 weeks also improved HbA1c and HDL. In children and adolescents with T1D, exercise reduces BMI, glycemic markers, and HDL, while HIIT enhances VO_2max/peak_ in adults with T1D. Overall, it can be concluded that exercise training effectively improves BMI, glycemic markers, and cardiorespiratory fitness in T1D patients, with CET and AET offering more beneficial impacts, especially in longer-term durations and children and adolescents.

## Figures and Tables

**Table 1:** Characteristics of participants and interventions.

Source, Year	Sample Size (Sex)	Intervention	Participant Characteristics	Age [Years]	BMI [kg/m^2^]	Ex Program Duration, Type, and Frequency	Intervention Protocol	Supervision	Outcomes
Campaigne et al., 1984 [36]	19 (7 F, 12 M)	AET Con	T1D, children	AET: 9.0 ± 0.47 Con: 8.5 ± 0.57	NG	12 w, AET, 3 d/w	AET: 30 min of 160 bpm running, movement to music. Con: no exercise	ND	HbA1c, FBG, VO_2max_
Yki-Jarvinen et al., 1984 [37]	13	AET Con	T1D, adolescent	AET: 26 ± 2.64 Con: 24 ± 2.44	NG	6 w, AET, 4 d/w	AET: cycle ergometer, 60 min, 150–160 bpm. Con: no exercise	ND	HbA1c, FBG, TCH, TG, HDL
Campaigne et al., 1985 [38]	14 (8 F, 6 M)	AET Con	T1D, adolescent	AET: 16 ± 1 Con: 15 ± 0.4	NG	12 w, AET 3 d/w	AET: 45 min aerobic movement to music at 80% HR_max_. Con: routine physical activity	Supervised	LBM, VO_2max,_ TCH, TG, HDL, LDL
Landt et al., 1985 [39]	15	AET Con	T1D, adolescent	AET: 16.1 ± 0.8 Con: 15.9 ± 0.3	NG	12 w, AET, 3 d/w	AET: 45 min, 80–85% HR_max_ Con: usual routines	Supervised	LBM, VO_2max_
Wallberg Henriksson et al., 1986 [21]	13	AET Con	T1D, adult	AET: 36 ± 2 Con: 35 ± 2	AET: 21.7 ± 0.9 Con: 22.0 ± 0.4	20 w, AET, 20 min daily	AET: Bicycle, 60–90% VO_2peak_. Con: same treatment except for the daily 20 min training sessions	Unsupervised	HbA1c, VO_2max_, TCH, TG, HDL, LDL
Stratton et al., 1987 [40]	16	CET Con	T1D, adolescent	CET: 15.1 ± 1.2 Con: 15.5 ± 0.9	NG	8 w, AET, 3 d/w	CET: 30–45 min, 2 d/w: jogging or cycling + 1 d/w: basketball, recreational swimming, or resistance exercise machines	Supervised	HbA1c, FBG, TCH, TG, HDL
Huttunen et al., 1989 [41]	34 (20 M, 14 F)	AET Con	T1D, children and adolescent	8–17	NG	13 w, AET, 1 d/w	AET: 60 min, 72% HR_max_ Con: equal amount of time on activities that did not require physical effort	Supervised	HbA1c, FBG, VO_2max_
Durak et al., 1990 [42]	8 (M)	RET Con	T1D, adult	RET: 31.5 ± 2 Con: 30.5 ± 5	NG	10 w, RET, 3 d/w	RET: 10 upper-body exercises and 4 lower-body exercises with free weight and resistance exercise machines, rest: 3–7 sets, <12 reps, 30 s to 2 min between sets	supervised	HbA1c, FBG, BFP, Strength, TCH, TG, HDL, LDL
Laaksonen et al., 2000 [43]	42	AET Con	T1D adult	AET: 31.7 ± 5.8 Con: 29.8 ± 6.4	AET: 24.4 ± 1.9 Con: 24.4 ± 2.2	12–16 w, AET, 3–5 d/w	AET: 20–30 min at 50–60% VO_2peak_ Con: routine physical activity	Unsupervised	HbA1c, FBG, BMI, BFP, VO_2max_, TCH, TG, HDL, LDL
Fuchsjager-Mayri et al., 2002 [44]	21	AET Con	T1D, adult	AET: 42 ± 10 Con: 33 ± 11	AET: 24.7 ± 0.9 Con: 26.7 ± 1.4	4 months, AET 2–3 d/w	AET: 60 min stationary cycling at 60–70% HR_max_	supervised	HbA1c, BMI, VO_2max_, TCH, TG, HDL, LDL
Roberts et al., 2002 [45]	24	AET + ANET Con	T1D, adolescent	14.0 ± 1.2	AET + ANET: 21.4 ± 2.5 Con: 19.4 ± 2.4	12 w, AET + ANET 3 d/w	AET + ANET: 45 min, AET: ANET, 7:3 HR ≥160 bpm. Con: without any training	Supervised/unsupervised	HbA1c, BMI
Heyman et al., 2007 [46]	16	CET Con	T1D, adolescent	CET: 15.9 ± 1.5 Con: 16.3 ± 1.2	CET: 24.5 ± 4.6 Con: 25.1 ± 3.9	6 months, CET, 3 d/w	CET: AET: intermittent workloads at 80–90% of HRR + RET, AET: RET of 2:1. Con: monitoring physical activity	Supervised/unsupervised	TCH, TG, HDL, LDL
Newton et al., 2009 [47]	74	AET Con	T1D, adolescent	14.4 ± 2.37	NG	12 w, AET, daily	AET: at least 10,000 steps daily. Con: received standard care	ND/ unsupervised	HbA1c, BP
Salem et al., 2010 [48]	196 (75 M 121 F)	CET 1, CET 2 Con	T1D, adolescent	CET 1: 14.7 ± 2.2 CET 2: 14.5 ± 2.4 Con: 15 ± 2.35	NG	6 months, CET 1: 1 d/w, CET 2: 3 d/w	CET: AET: 20 min at 65–85% HR_max_ cycling / treadmill + ANET: 1–2 min at 85–95% HR_max_ + RET: DeLorme technique: 3 sets at 50–70% of 10-RM, rests: 2 min between sets + free strength and endurance exercises: 10 min + neuromuscular exercises: 5 min + flexibility: 5 min. Con: usual routines	supervised	HbA1c, BMI, TCH, TG, HDL, LDL
Dhooge et al., 2011 [17]	16	CET Con	T1D, adolescent	CET: 13.66 ± 5.98 Con: 12.86 ± 4.64	CET: 21.41 ± 12.15 Con: 19.12 ± 6.70	20 w, 2 d/w	CET: AET: 40 min at 60–75% of HR_max_ + RET: 2–3 sets, 10–15 reps, 12–20 1-RM Con: routine physical activity	Supervised	HbA1c, BMI, VO_2max_, Strength
Wong et al., 2011 [49]	23	AET Con	T1D, children and adolescent	AET: 11.62 ± 2.12 Con: 12.77 ± 1.79	AET:17.43 ± 2.61 Con: 18.84 ± 2.67	12 w, AET, 3 d/w	AET: Home-based, 40–60% HRR, 10–30 min Con: no exercise or self-directed exercise	ND	HbA1c
Maggio et al., 2012 [50]	27	AET Con	T1D, children	AET: 10.5 ± 2.0 Con: 10.5 ± 2.9	AET: 18.5 ± 2.4 Con: 18.6 ± 2.3	9 months, weight-bearing activities 2 d/w	AET: 90 min of weight-bearing activities at 140 bpm Con: were relatively inactive	Supervised	BMI, LBM
Tunar et al., 2012 [51]	31	Pilates Con	T1D, adolescent	Pilates: 14.2 ± 2.2 Con: 14.3 ± 1.8	NG	12 w, Pilates, 3 d/w	Pilates: 45 min. Con: usual activities	Supervised	HbA1c, TCH, TG, HDL, LDL
Gusso et al., 2017 [52]	50 (24 F, 26 M)	CET Con	T1D, adolescent	CET: 15.6 ± 1.3 Con: 15.5 ± 0.9	CET: 23.53 ± 1.77 Con: 24.6 ± 2.94	20 w, CET, 3–4 d/w	CET: AET: 40 min at 85% HR_max_ + RET: weight training and core exercises. Con: did not participate in the exercise program	Supervised	HbA1c, BMI, BFP, VO_2max_, BP.
Boff et al., 2019 [16]	27	HIIT MCT Con	T1D, adult	HIIT: 26.1 ± 7.8 MCT: 23.7 ± 5.8 Con: 20.8 ± 2.6	HIIT: 23.2 ± 2.4 MCT: 24.1 ± 2.0 Con: 22.7 ± 2.6	8 w, HIIT or MCT, 3 d/w	HIIT: 50–85% HR_max_ MCT: 60–65% HR_max_ Con: general lifestyle recommendations, walk at least 3 d/w for 30 min	Supervised	HbA1c, FBG, VO_2max_, TCH, TG, HDL, LDL, BP
Petschnig et al., 2020 [53]	21	RET Con	T1D, children	RET: 11.00 ± 0.8 Con: 11.3 ± 0.7	RET: 19.26 ± 2.4 Con: 19.55 ± 4.2	32 w, RET, 2 d/w	RET: circuit, 20–40 min, each station: 25–40 s, ≥30% 1-RM, rest between cycles: 180 s, and between stations: 40–30 s. Con: No exercise	supervised	HbA1c, Strength
Lee et al., 2020 [18]	22	HIIT Con	T1D, adult	HIIT: 40.5 ± 10.0 Con: 46.1 ± 10.5	HIIT: 29.0 ± 2.1 Con: 31.6 ± 3.4	12 w, HIIT, 3 d/w	HIIT: 33 min, 4 × 4 min, 85–95% HR_max_, 3 min recovery intervals at 50–70% HR_max_	Supervised/ unsupervised	HbA1c, BMI, BFP, VO_2peak_, Strength, TCH, TG, HDL, LDL, BP
Alarcon-Gomez et al., 2021 [14,15]	19	HIIT Con	T1D, adult	HIIT: 38 ± 5.5 Con: 35 ± 8.2	HIIT: 25.1 ± 0.4 Con: 25.2 ± 0.8	6 w, HIIT, 3 d/w	HIIT: 12–20 bouts of 30 s at 85% PPO, 1 min recovery at 40% PPO Con: routine lifestyle and dietary intakes	supervised	First: BFP, LBM, VO_2max_, FBG Second: HbA1c, BMI
Mohammad et al., 2021 [19], and 2023 [20]	20 (M)	Football Con	T1D, adolescent	Football: 17.8 ± 0.42 Con: 14.4 ± 2.0	Football: 23.71 ± 3.34 Con: 23.46 ± 1.96	12 w, football, 2 d/w	Football: 90 min at 80% HR_max_. Con: usual activities	NG	First: HbA1c, FBG, TCH, TG, HDL, LDL, BP Second: BMI, VO_2max_
Nazari et al., 2023 [54]	40	Concurrent Con	T1D, children and adolescent	Concurrent: 11.22 ± 1.90 Con:11.00 ± 2.67	Concurrent: 18.96 ± 4.11 Con: 17.28 ± 1.87	16 w, concurrent, 3 d/w	Concurrent: AET: 50–75% HR_max_ + Pilates 2–3 sets of 8–12 reps, rest: 30 s between the sets. Con: no exercise	Supervised	HbA1c, FBG

Abbreviations: HIIT: high intensity interval training; MCT: moderate continuous training; AET: aerobic exercise training; RET: resistance exercise training; CET: combination exercise training; ANET: anaerobic exercise training; T1D: type 1 diabetes; F: female; M: male; PPO: peak power output; BFP: body fat percentage; LBM: lean body mass; VO_2max_: maximum oxygen uptake; VO_2peak_: peak oxygen uptake; FBG: fasting blood glucose; BMI: body mass index; HbA1c: glycated hemoglobin; LDL: low-density lipoprotein; HDL: high-density lipoprotein; TG: triglyceride; TCH: total cholesterol; BP: blood pressure; HR_max_: maximum heart rate; HRR: heart rate reserve; bpm: beats per minute; s: second; min: minute; NG: not given.

## Data Availability

The original contributions presented in this study are included in the article/Supplementary Material. Further inquiries can be directed to the corresponding authors.

## Supplementary Materials

The following supporting information can be downloaded at: https://www.mdpi.com/article/10.3390/healthcare13030246/s1: Table S1. Search strategy; Table S2. Risk of bias assessment; Figure S1. Forest plot of the effects of exercise training versus control on TG; Figure S2. Forest plot of the effects of exercise training versus control on TC; Figure S3. Forest plot of the effects of exercise training versus control on LDL; Figure S4. Forest plot of the effects of exercise training versus control on HDL; Figure S5. Forest plot of the effects of exercise training versus control on SBP; Figure S6. Forest plot of the effects of exercise training versus control on DBP; Figure S7. Forest plot of the effects of exercise training versus control on VO_2max/peak_.
