# Supplementary material for: The Effects of Exercise Training on Body Composition and Cardiometabolic Risk Factors in Type 1 Diabetes Mellitus: A Systematic Review and Meta-Analysis
Source: Healthcare (Basel). 2025 Jan 26;13(3):246. doi: 10.3390/healthcare13030246 (PMC11816365; doi:10.3390/healthcare13030246)
Supplement: Supplementary file 1 [file healthcare-13-00246-s001.zip › healthcare-3330312-supplementary.pdf]

Supplementary Tables and Figures

Supplementary Table 1. Search strategy

PubMed	(("exercise"[MeSH Terms] OR "exercise"[All Fields] OR "exercises"[All Fields] OR "exercise therapy"[MeSH Terms] OR ("exercise"[All Fields] AND "therapy"[All Fields]) OR "exercise therapy"[All Fields] OR "exercising"[All Fields] OR "exercise s"[All Fields] OR "exercised"[All Fields] OR "exerciser"[All Fields] OR "exercisers"[All Fields] OR "exercise training"[All Fields] OR "physical activity"[All Fields]) AND ("Type1 diabetes"[All Fields] OR "Insulin-Dependent"[All Fields] OR "Insulin-Dependent Diabetes Mellitus"[All Fields] OR "Type 1 Diabetes Mellitus"[All Fields] OR "Insulin-Dependent Diabetes Mellitus 1"[All Fields] OR ("diabetes mellitus, type 1"[MeSH Terms] OR "Type 1 Diabetes Mellitus"[All Fields] OR "iddm"[All Fields])) AND ("random allocation"[MeSH Terms] OR ("random"[All Fields] AND "allocation"[All Fields]) OR "random allocation"[All Fields] OR "randomization"[All Fields] OR "randomized"[All Fields] OR "random"[All Fields] OR "randomisation"[All Fields] OR "randomisations"[All Fields] OR "randomise"[All Fields] OR "randomised"[All Fields] OR "randomising"[All Fields] OR "randomizations"[All Fields] OR "randomize"[All Fields] OR "randomizes"[All Fields] OR "randomizing"[All Fields] OR "randomness"[All Fields] OR "randoms"[All Fields] OR ("random allocation"[MeSH Terms] OR ("random"[All Fields] AND "allocation"[All Fields]) OR "random allocation"[All Fields] OR "randomization"[All Fields] OR "randomized"[All Fields] OR "random"[All Fields] OR "randomisation"[All Fields] OR "randomisations"[All Fields] OR "randomise"[All Fields] OR "randomised"[All Fields] OR "randomising"[All Fields] OR "randomizations"[All Fields] OR "randomize"[All Fields] OR "randomizes"[All Fields] OR "randomizing"[All Fields] OR "randomness"[All Fields] OR "randoms"[All Fields]) OR "randomly"[All Fields] OR "randomized control"[All Fields] OR "randomized control trial"[All Fields] OR "randomized clinical trial"[All Fields])) AND (humans[Filter])
Scopus	(TITLE-ABS-KEY (exercise OR "exercise training" OR "physical activity") AND TITLE-ABS-KEY ("Type1 diabetes" OR "Insulin-Dependent" OR "Insulin-Dependent Diabetes Mellitus" OR "Type 1 Diabetes Mellitus" OR "Insulin-Dependent Diabetes Mellitus 1" OR iddm) AND TITLE-ABS-KEY (random OR randomized OR randomly OR "randomized control" OR "randomized control trial" OR "randomized clinical trial"))
WOS	((TS=(exercise or "exercise training" or "physical activity")) AND TS=("Type1 diabetes" or "Insulin-Dependent" or "Insulin-Dependent Diabetes Mellitus" or "Type 1 Diabetes Mellitus" or "Insulin-Dependent Diabetes Mellitus 1" or IDDM)) AND TS=(random or randomized or randomly or "randomized control" or "randomized control trial" or "randomized clinical trial")

Supplementary Table 2. Risk of bias assessment

	Eligibility Criteria	Random Allocation	Allocation Concealment	Baseline Comparability	Blinding of Assessors	Outcome Measurement	Intention-to-Treat Analysis	Between-Group Comparisons	Point and Variability Measures
2021 Alarcon-Gomez et al.	+	+	+	_	_	+	_	+	+
Alarcon-Gomez et al. 2021	+	+	+	_	_	+	_	+	+
Boff et al. 2019	+	+	+	+	+	_	_	+	+
1984 Campaigne et al.	_	+	_	+	_	+	_	+	+
1984 Campaigne et al.	_	+	_	+	_	+	_	+	+
D hooge et al. 2011	_	+	+	+	+	_	_	+	+
Durak et al. 1990	_	+	_	_	_	+	_	+	+
Fuchsjager –Mayri et al. 2002	+	+	_	+	_	_	_	+	+
2017 Gusso et al.	_	+	+	+	+	+	_	+	+
2007 Heyman et al.	_	+	_	+	_	+	_	+	+
Huttunen et al. 1989	_	+	_	_	_	+	_	+	+
Laaksonen et al. 2000	+	+	_	+	_	_	_	+	+
Landt et al. 1985	_	+	_	_	_	+	_	+	+
Lee et al. 2020	+	+	+	+	_	_	+	+	+
Maggio et al. 2012	+	+	_	+	+	+	+	+	+
Mohammad et al. 2021	+	+	_	+	+	+	_	+	+
Mohammad et al. 2023	_	+	_	+	_	+	_	+	+
Nazari et al. 2023	+	+	_	_	_	+	_	+	+
Newton et al. 2009	_	+	_	+	+	+	+	+	+
Petschnig et al. 2020	+	+	_	+	_	_	_	+	+
Robert er al. 2002	_	+	_	_	_	+	_	+	+
Salem et al. 2010	+	+	_	+	_	_	_	+	+
Stratton et al.1987	_	+	_	+	_	+	_	+	+
Tunar et al. 2012	_	+	_	_	+	+	_	+	+
Wallberg Henriksson et al. 1986	+	+	_	_	_	_	_	+	+
Wong et al. 2011	_	+	_	+	_	_	_	+	+
Yki-Jarvinen et al. 1984	_	+	_	_	_	+	_	+	+

Model	Study name	Difference in means	Standard error	Variance	Lower limit	Upper limit	Z-Value	p-Value
	Boff et al. (2019) a	-26.570	22.621	511.700	-70.906	17.766	-1.175	0.240
	Boff et al. (2019) b	-14.170	46.184	2132.972	-104.689	76.349	-0.307	0.759
	Campaigne et al. (1985)	5.200	26.322	692.828	-46.389	56.789	0.198	0.843
	Heyman et al. (2007)	-1.780	14.399	207.325	-30.001	26.441	-0.124	0.902
	Laaksonen et al. (2000)	-25.690	14.118	199.319	-53.361	1.981	-1.820	0.069
	Lee at al. (2020)	35.400	22.736	516.929	-9.162	79.962	1.557	0.119
	Mohammed et al. (2021)	37.240	6.383	40.738	24.730	49.750	5.835	0.000
	Salem et al. (2010) a	-13.700	8.857	78.450	-31.060	3.660	-1.547	0.122
	Salem et al. (2010) b	-41.800	7.986	63.782	-57.453	-26.147	-5.234	0.000
	Stratton et al. (1987)	-40.100	45.080	2032.235	-128.456	48.256	-0.890	0.374
	Tunar et al. (2012)	13.000	20.921	437.678	-28.004	54.004	0.621	0.534
	Wallberg Henriksson et al. (1986)	7.080	12.146	147.528	-16.726	30.886	0.583	0.560
	Yki-Jarvinen et al. (1984)	-23.910	18.400	338.553	-59.973	12.153	-1.299	0.194
Random		-5.379	10.034	100.671	-25.044	14.287	-0.536	0.592

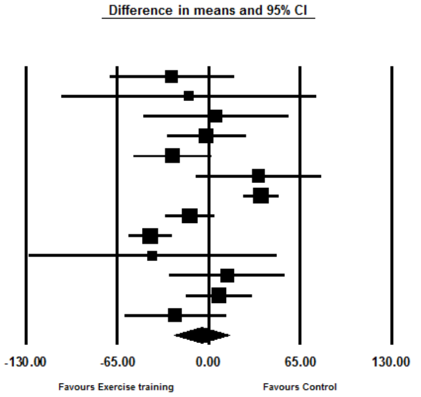

Supplementary Figure 1. Forest plot of the effects of exercise training versus control on TG. Data are reported as WMD (95% confidence limits). WMD: weighted mean difference.

Model	Study name	Difference in means	Standard error	Variance	Lower limit	Upper limit	Z-Value	p-Value
	Boff et al. (2019) a	-5.030	15.800	249.633	-35.997	25.937	-0.318	0.750
	Boff et al. (2019) b	-8.500	31.097	967.046	-69.450	52.450	-0.273	0.785
	Campaigne et al. (1985)	-12.600	26.226	687.786	-64.001	38.801	-0.480	0.631
	Heyman et al. (2007)	6.200	12.061	145.466	-17.439	29.839	0.514	0.607
	Laaksonen et al. (2000)	-11.980	12.554	157.595	-36.585	12.625	-0.954	0.340
	Lee et al. (2020)	7.700	12.693	161.119	-17.178	32.578	0.607	0.544
	Mohammed et al. (2021)	-5.800	6.758	45.668	-19.045	7.445	-0.858	0.391
	Salem et al. (2010) a	-17.760	10.745	115.465	-38.821	3.301	-1.653	0.098
	Salem et al. (2010) b	-51.300	6.169	38.062	-63.392	-39.208	-8.315	0.000
	Stratton et al. (1987)	11.600	30.737	944.784	-48.644	71.844	0.377	0.706
	Tunar et al. (2012)	-0.400	16.422	269.698	-32.587	31.787	-0.024	0.981
	Wallberg Henriksson et al. (1986)	-10.440	16.861	284.277	-43.486	22.606	-0.619	0.536
	Yki--Jarvien et al. (1984)	-27.060	12.451	155.028	-51.464	-2.656	-2.173	0.030
Random		-11.822	6.917	47.846	-25.380	1.735	-1.709	0.087

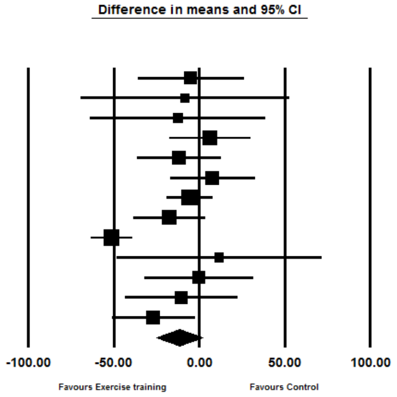

Supplementary Figure 2. Forest plot of the effects of exercise training versus control on TC. Data are reported as WMD (95% confidence limits). WMD: weighted mean difference.

Model	Study name	Difference in means	Standard error	Variance	Lower limit	Upper limit	Z-Value	p-Value
	Boff et al. (2019) a	3.480	13.585	184.552	-23.146	30.106	0.256	0.798
	Boff et al. (2019) b	-19.720	27.714	768.061	-74.038	34.598	-0.712	0.477
	Campaigne et al. (1985)	-11.400	24.635	606.895	-59.684	36.884	-0.463	0.644
	Heyman et al. (2007)	2.710	10.230	104.643	-17.339	22.759	0.265	0.791
	Laaksonen et al. (2000)	-7.740	9.261	85.771	-25.892	10.412	-0.836	0.403
	Lee et al. (2020)	-11.600	11.602	134.597	-34.339	11.139	-1.000	0.317
	Mohammed et al. (2021)	24.000	8.669	75.149	7.009	40.991	2.769	0.006
	Salem et al. (2010) a	-12.700	7.774	60.440	-27.937	2.537	-1.634	0.102
	Salem et al. (2010) b	-30.200	6.886	47.415	-43.696	-16.704	-4.386	0.000
	Tunar et al. (2012)	-6.400	8.500	72.253	-23.060	10.260	-0.753	0.451
	Wallberg-Henriksson et al. (1986)	13.150	15.908	253.078	-18.030	44.330	0.827	0.408
Random		-4.887	5.559	30.908	-15.784	6.009	-0.879	0.379

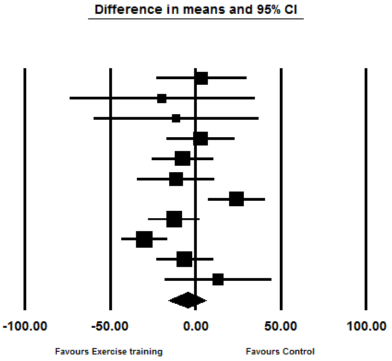

Supplementary Figure 3. Forest plot of the effects of exercise training versus control on LDL. Data are reported as WMD (95% confidence limits). WMD: weighted mean difference.

Model	Study name	Difference in means	Standard error	Variance	Lower limit	Upper limit	Z-Value	p-Value
	Boff et al. (2019) a	-1.550	4.621	21.350	-10.606	7.506	-0.335	0.737
	Boff et al. (2019) b	-5.020	5.141	26.427	-15.096	5.056	-0.977	0.329
	Campaigne et al. (1985)	-1.600	5.325	28.358	-12.037	8.837	-0.300	0.764
	Heyman et al. (2007)	3.870	6.091	37.102	-8.068	15.808	0.635	0.525
	Laaksonen et al. (2000)	0.390	4.306	18.541	-8.050	8.830	0.091	0.928
	Lee et al. (2020)	7.800	7.701	59.309	-7.294	22.894	1.013	0.311
	Mohammed et al. (2021)	-13.920	5.039	25.395	-23.797	-4.043	-2.762	0.006
	Salem et al. (2010) a	8.800	2.670	7.128	3.567	14.033	3.296	0.001
	Salem et al. (2010) b	11.600	1.196	1.429	9.257	13.943	9.702	0.000
	Stratton et al. (1987)	4.600	4.705	22.141	-4.623	13.823	0.978	0.328
	Tunar et al. (2012)	-3.000	4.696	22.053	-12.204	6.204	-0.639	0.523
	Wallberg Henriksson et al. (1986)	-2.320	12.759	162.793	-27.327	22.687	-0.182	0.856
	Yki-Jarvinen et al. (1984)	3.100	5.922	35.067	-8.506	14.706	0.523	0.601
Random		1.505	2.443	5.967	-3.283	6.292	0.616	0.538

Statistics for each study

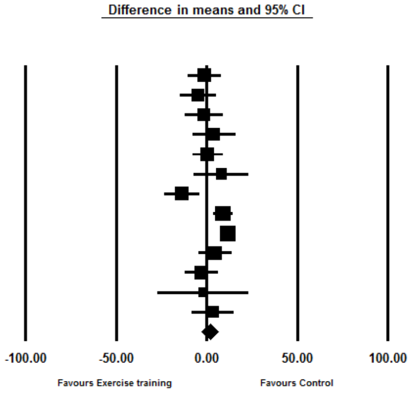

Supplementary Figure 4. Forest plot of the effects of exercise training versus control on HDL. Data are reported as WMD (95% confidence limits). WMD: weighted mean difference.

Model	Study name	Difference in means	Standard error	Variance	Lower limit	Upper limit	Z-Value	p-Value
	Boff et al. (2019) a	3.700	4.591	21.076	-5.298	12.698	0.806	0.420
	Boff et al. (2019) b	-5.600	4.733	22.403	-14.877	3.677	-1.183	0.237
	Gusso et al. (2017)	0.800	2.512	6.308	-4.123	5.723	0.319	0.750
	Lee et al. (2020)	6.600	5.831	33.995	-4.828	18.028	1.132	0.258
	Newton et al. (2009)	2.130	2.829	8.005	-3.415	7.675	0.753	0.452
Random		1.266	1.571	2.469	-1.814	4.346	0.806	0.420

Statistics for each study

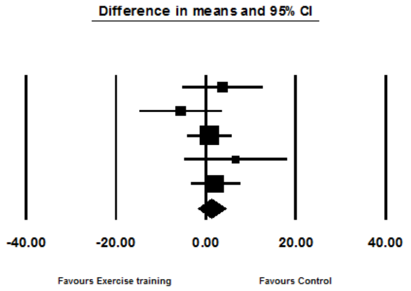

Supplementary Figure 5. Forest plot of the effects of exercise training versus control on SBP. Data are reported as WMD (95% confidence limits). WMD: weighted mean difference.

Model	Study name	Difference in means	Standard error	Variance	Lower limit	Upper limit	Z-Value	p-Value
	Boff et al. (2019) a	6.700	4.741	22.479	-2.593	15.993	1.413	0.158
	Boff et al. (2019) b	1.700	3.807	14.494	-5.762	9.162	0.447	0.655
	Gusso et al. (2017)	-0.500	2.332	5.438	-5.070	4.070	-0.214	0.830
	Lee et al. (2020)	-2.100	4.437	19.689	-10.797	6.597	-0.473	0.636
	Newton et al. (2009)	1.660	1.788	3.196	-1.844	5.164	0.929	0.353
Random		1.114	1.230	1.513	-1.297	3.524	0.905	0.365

Statistics for each study

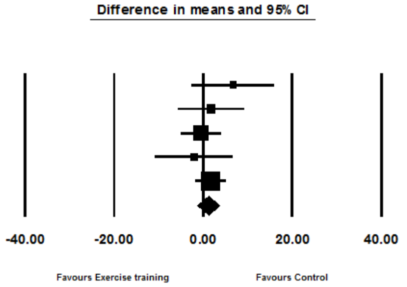

Supplementary Figure 6. Forest plot of the effects of exercise training versus control on DBP. Data are reported as WMD (95% confidence limits). WMD: weighted mean difference.

Model	Study name	Difference in means	Standard error	Variance	Lower limit	Upper limit	Z-Value	p-Value
	Alarcon-Gomez et al. (2021)	3.100	2.121	4.498	-1.057	7.257	1.462	0.144
	Boff et al. (2019) a	6.420	1.264	1.598	3.942	8.898	5.078	0.000
	Boff et al. (2019) b	3.380	1.632	2.663	0.181	6.579	2.071	0.038
	Campaigne et al. (1984)	1.070	2.812	7.905	-4.441	6.581	0.381	0.704
	Campaigne et al. (1985)	2.790	4.942	24.426	-6.897	12.477	0.565	0.572
	Huttunen et al. (1989)	2.500	2.763	7.632	-2.915	7.915	0.905	0.366
	Laaksonen et al. (2000)	1.300	2.303	5.305	-3.214	5.814	0.564	0.572
	Landt et al. (1985)	4.700	4.586	21.030	-4.288	13.688	1.025	0.305
	Lee et al. (2020)	1.200	2.240	5.016	-3.190	5.590	0.536	0.592
	Mohammed et al. (2023)	1.170	0.795	0.632	-0.388	2.728	1.472	0.141
	Wallberg Henriksson et al. (1986)	2.500	2.107	4.440	-1.630	6.630	1.186	0.235
Random		2.769	0.697	0.486	1.402	4.135	3.971	0.000

Statistics for each study

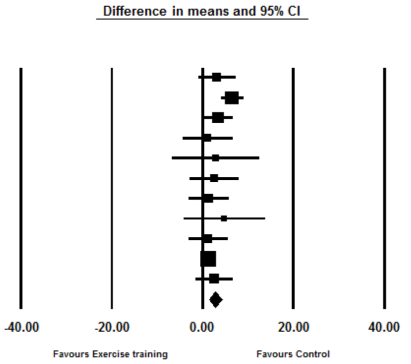

Supplementary Figure 7. Forest plot of the effects of exercise training versus control on **$VO_{2max/peak}$.** Data are reported as WMD (95% confidence limits). WMD: weighted mean difference.
